# Can Accelerated Aging Procedures Predict the Long Term Behavior of Polymers Exposed to Different Environments?

**DOI:** 10.3390/polym13162688

**Published:** 2021-08-12

**Authors:** Mariaenrica Frigione, Alvaro Rodríguez-Prieto

**Affiliations:** 1 Department of Engineering for Innovation, University of Salento, Prov. le Lecce-Monteroni, 73100 Lecce, Italy; 2 Department of Manufacturing Engineering, Universidad Nacional de Educación a Distancia (UNED), 28040 Madrid, Spain; alvaro.rodriguez@ind.uned.es; 3 Department of Industrial Inspection and Technical Assistance, SGS Tecnos, 28042 Madrid, Spain

**Keywords:** chemical aging, durability, on-field tests, natural exposure, polymer accelerated aging

## Abstract

During their useful life, polymers are subject to degradation processes due to exposure to specific environmental conditions over long times. These processes generally lead to changes, almost always irreversible, of properties and performances of polymers, changes which would be useful to be able to predict in advance. To meet this need, numerous investigations have been focused on the possibility to predict the long-term performance of polymers, if exposed to specific environments, by the so called “accelerated aging” tests. In such procedures, the long-term behavior of polymeric materials is typically predicted by subjecting them to cycles of radiations, temperatures, vapor condensation, and other external agents, at levels well above those found in true conditions in order to accelerate the degradation of polymers: this can produce effects that substantially deviate from those observable under natural exposure. Even following the standard codes, different environmental parameters are often used in the diverse studies, making it difficult to compare different investigations. The correlation of results from accelerated procedures with data collected after natural exposure is still a debated matter. Furthermore, since the environmental conditions are a function of the season and the geographical position, and are also characteristic of the type of exposure area, the environmental parameters to be used in accelerated aging tests should also consider these variables. These and other issues concerning accelerated aging tests applied to polymers are analyzed in the present work. However, bearing in mind the limitations of these practices, they can find useful applications for rating the durability of polymeric materials.

## 1. Introduction

Synthetic and natural polymers, as any other material, experience some type and level of degradation during their service-life, resulting in an alteration of their (mechanical, thermal, optical, functional, other) properties, thus affecting their performance and shortening their useful life-cycle [1,2,3,4,5,6,7]. The long term behavior of a polymeric material is influenced by its chemical nature, the process conditions used to manufacture and/or to apply it, on the load regime to which it is subjected and, mostly, on the kind and level of environmental exposure in which the material operates, i.e., by its service conditions. The same polymer, in fact, can perform differently if exposed to different conditions, for instance if exposed indoor or outdoor, in rural or industrial environment.

The environmental factors that most affect the characteristics and properties of polymers are also those most frequently encountered in the common service conditions, i.e., liquid or vapor water, aqueous solutions, oxygen, UV/solar radiations, variations in temperature, chemicals, acid or alkaline environments, fuels, pollutants. Any specific application, in which polymeric materials perform a certain task, are characterized by distinctive service conditions, as for examples: automotive components, typically made in thermoplastic polymers, are exposed to medium/high temperatures (close to engine: over 100–130 °C), water/humidity, radiations, fuels, chemicals; elastomeric polymers composing tires are exposed to oxygen and medium temperatures, water/humidity, fuel, chemicals, mechanical (cyclic) stresses; thermosetting matrices to manufacture fiber reinforced polymers (FRP) applied in aeronautical fields are subjected to radiations and exposure to thermal cycles, water/humidity, ice, fuel, chemicals, mechanical (cyclic) stresses. Polymers can be even accidentally exposed to extreme (and unpredictable) environments, such as: fire, earthquake, floods, explosive blasts, nuclear radiations.

In real conditions, many environmental agents can be present simultaneously, as reported in the examples previously introduced: the contemporary presence of more environmental agents and, possibly, of mechanical stresses makes synergistic actions possible. Moreover, the synchronicity of some climatic factors (for instance: UV radiations and temperature) can dramatically affect the durability of a polymer, explaining why a faster degradation takes place in polymers outdoor exposed in places characterized by a high UV irradiation and elevated temperatures [8].

Furthermore, the common outdoor conditions are not predictable neither constant, depending (not exclusively) on the place of exposure (i.e., on altitude, latitude, distance from sea, etc.), on the season of the year and on the variable weather conditions: thus, their effects on the properties of polymers are equally unpredictable. As an example, the percentage of UV radiations that reaches the surface of a polymeric component depends in a complex way on humidity and/or rain, cloudless exposure period, direction and speed of wind, latitude and angle of incidence of the radiation on the polymeric surface. The degradation taking place in a polymer depends not only on the service exposure conditions, therefore on the effect that any single environmental agent can exert on the polymer, but also on the level and the time of exposure to such service conditions and on the load regime.

Depending on the nature of the external detrimental agent, polymer degradation can be classified in oxidation and photo-oxidative degradation, thermal and thermo-oxidative degradation, ozone-induced degradation, mechanical and chemical degradation, hydrolytic degradation, just to mention the main chemical processes caused by the most commonly encountered agents. The effects of these agents on polymer’s properties are often permanent, since the majority of the mentioned processes are characterized by not reversible reactions (for instance: breaking or formation of covalent bonds inside the macromolecules).

Degradation is an inevitable process for any material, and also for polymers: it cannot be arrested unless it is interrupted in some manner, for instance isolating the material from the environments that have proved to be aggressive and stopping any mechanical stress. However, for any application involving polymers, it is much more important to assess how long degradation must proceed to become manifest, resulting in a decay in properties that can significantly affect the end-use properties, i.e., its long-term durability in a specific environment.

In a systematic complete study of the service durability of a polymeric material, the effects of any external agent characterizing the specific application of the polymer must be analyzed and quantified. However, the analysis of the influence of any single agent on the properties of the polymeric material does not guarantee to outline the durability of the material subject to a cooperating multitude of environmental factors, since the effects may not be additive and, as underlined, synergistic actions cannot be excluded.

The natural aging of a polymeric material is the only approach able to provide reliable results. However, on field exposure requires long analysis times (in the order of decades) in order to collect meaningful data and, then, draw trustworthy conclusions. A fascinating alternative is represented by the accelerated aging procedures. As the name suggests, these procedures are intended to provide previsions on the durability of a polymeric material, i.e., they would predict its long-term properties and characteristics, amplifying at selected levels one or more environmental parameters characterizing the true exposure. In this way, employing proper environmental chambers or specific devices, these procedures try to simulate in a very short time the effects on the polymer of the same parameters due to a long exposure, thus reducing the long time required to the durability assessment of the polymeric material. Accelerated procedures are also very attractive to producers of new materials for a quick assessment of their long-term performance, with a consequent reduction in the times required for their placing on the market. On the other hand, a critical limit of accelerated aging relies on the use of environmental factors much higher than those achievable on field, being able to cause unrealistic processes that would never occur in true conditions [9].

This manuscript aims at providing an overview on this field of research since, despite a large number of works related to the use of accelerated procedures, there is a lack of fundamental knowledge on this subject: it is not guaranteed that these procedures are really capable to predict in a limited time of observation the long-time durability of polymeric materials. A review of recent papers appeared in literature dealing with the use of such procedures to reproduce what happens in true conditions is presented. From this analysis, the main issues that prevent a full and generalized reliability of these procedures, irrespective to the kind of polymer, are highlighted, i.e., issues that, at the present time, hinder a wider usefulness and exploitability of accelerated aging tests. Some suggestions and proposals of new routes to explore in order to improve the research in this field are, finally, presented.

## 2. Procedures and Standard Codes

**Figure 1:**
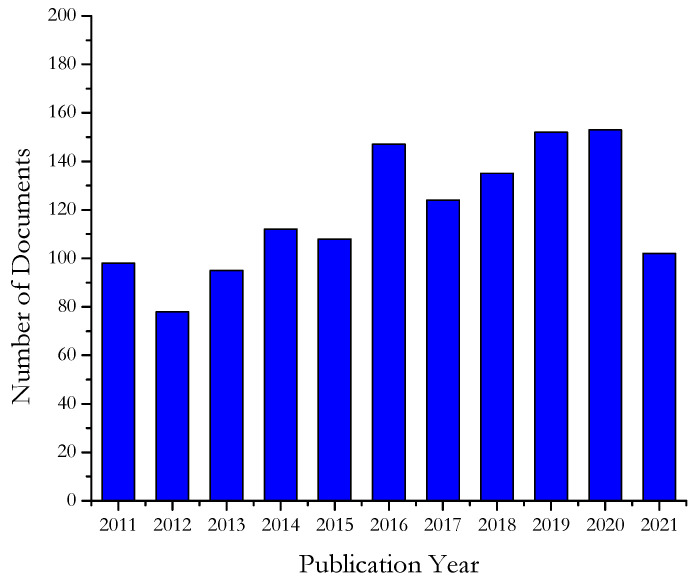
Papers published per year between January 2011 and June 2021 in journals, books, conference proceedings dealing with accelerated aging procedures for polymers found in the Scopus database.

The adoption of accelerated aging procedures to make reliable previsions of long-term durability of polymeric materials has been the subject of several papers by international research groups starting from the 80s of last century, even though few pioneering studies have been published since 1952. From Scopus database (to date 30 June), we counted 1304 documents, published in journals, books, conference proceedings in the last ten years (2011–2021) searching for “polymer accelerated aging” in document title, abstract, keywords, whose yearly distribution is illustrated in Figure 1.

It is worthwhile to underline that it is possible that not all the papers dealing with this topic are included in the previous list since “polymer accelerated aging” is not reported in their title, abstract or keywords. However, not all papers dealing with accelerated aging verify that such procedures are capable of predicting the long term behavior of polymers. Anyhow, the continuous production of the scientific community testifies how topical and challenging is this subject.

According to common standard procedures employed to perform accelerated aging tests, the polymeric material under investigation is exposed for a defined period of time or for a certain number of cycles to one (or more) detrimental agents at levels significantly higher than the conditions normally encountered on field, in order to intensify the effects of the same agent [10,11,12]. These experiments are typically carried out in proper climatic chambers, where the polymer specimens are exposed, in presence of oxygen, to high levels of humidity, temperature or variations in temperature, UV radiations, different (acid, alkaline, saline, etc.) atmospheres; possibly, heavy mechanical stresses can be used to reproduce also the load field to which the polymer is subjected. As a few examples of the selection of the intensified agents, in order to accelerate the effect of an immersion in water at ambient temperature, the polymer can be immersed in boiling water; when temperature is the environmental parameter under investigation, the material is exposed to much greater temperatures than those characteristic of the intended application. To accelerate the effects of exposure to natural sunlight, mirrors can be employed to both intensify the sun rays and follow its direction during the day.

The first step when designing an accelerated aging procedure is, therefore, the definition of the exposure parameters, these latter depending on the specific application [13]. The selection of the acceleration factors for the environmental agent under study plays a decisive role in the reliability of the tests [14]. These acceleration factors, in fact, must produce degradation mechanisms as close as possible to the degradation produced by the natural exposure and assure, at the same time, to reduce times for analysis, these two requirements being evidently in conflict.

**Figure 2:**
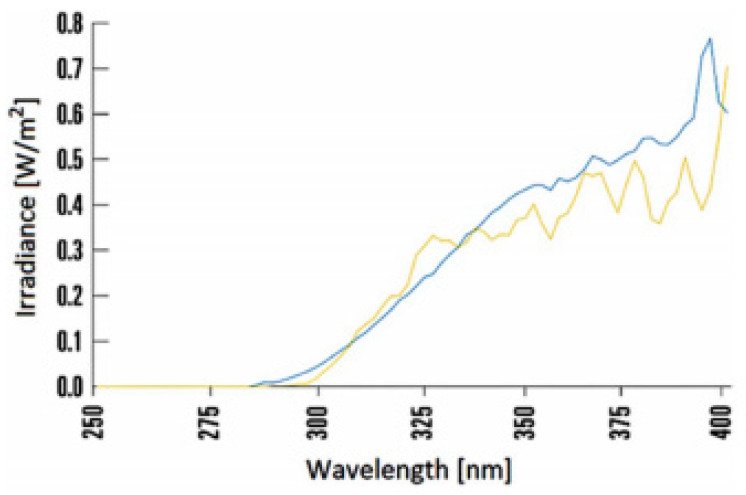
Comparison between the characteristic radiation spectrum of the xenon lamp (with a filter approximating the spectrum of sunlight), blue line; and the spectrum of sunlight, yellow line. Reprinted with permission [19].

Almost all the accelerated aging procedures employ radiations produced by different lamps (i.e., xenon, mercury, metal halide or carbon arc, fluorescent tube light sources) as accelerating agent [15], since it is well recognized that photo-chemical processes are the principal responsible for degradation of polymers. The degrading mechanisms taking place when a polymer is exposed to radiations depend on the irradiation wavelengths and radiative energy as well as on the chemical structure of the polymer [16]. It must be underlined that the fraction of UV-sunlight radiations reaching the earth after filtration of the atmosphere typically lies in the wavelength range 290–400 nm, representative of UV-B and UV-A radiations [15]. Moreover, ozone layer filters wavelengths in the range 200–290 nm, corresponding to UVC, and most of those in the range 290–320 nm, i.e., UVB: only UVA radiations, i.e., those in the range 320–400 nm, and a small amount of UVB reach the earth, both harmful for many polymers. Therefore, a device able to reproduce the solar natural exposure on the surface of a polymer should cut wavelengths lower than 290–320 nm threshold. UV-A fluorescent lamps, with a peak emission at 340 nm, are generally recommended to reproduce the damaging effect of sun radiations on most plastics intended for outdoor applications [17]. Some Authors reported that UV–A lamps (with emission peak at 340 nm and irradiance of 0.76 W/m^2^) are able to reproduce the radiations characteristic of a summer noon [7]. Similarly, a 340 nm UV-A lamp, with irradiance of 0.68 W/m^2^, is reported to be the best simulation of sunlight in the critical wavelength range where most of the polymer damage occurs, i.e., 365–295 nm [11]. It must be underlined that such lamps typically provide a lower acceleration factor than UV-B lamps, but more realistic test results. It has been recently recognized that the best choice, in terms of reproducibility of solar radiation, is offered by filtered xenon light source in the wavelength 290–400 nm range [15,18], as illustrated in Figure 2.

Referring to the effects of a moist/wet environment, distilled/deionized water is generally employed in the aging tests programs [20], while in true external conditions the materials are exposed to water, for instance rain water, not always comparable to pure water [21]. Furthermore, to accelerate the effect of exposure to water, immersion in boiling water has been frequently employed: this procedure is likely to cause unrealistic damages in polymers. The temperature, in fact, is often used as an acceleration factor to promote the degradation of the polymer under analysis in shorter times. This parameter, on the other hand, must not exceed, or even approach, the glass transition/melting temperature range of the polymer so as not to cause changes in materials that would not occur in real conditions (i.e., if they were naturally exposed).

To reproduce a saline atmosphere, salt spray cycles can be performed in a climatic chamber employing artificial seawater, for instance according to the proper code [22], or salt solution of a specific concentration, not necessarily capable of reproducing real conditions.

Taking into account the strategic nature that, especially in some sectors, represents the prediction of the long-term behavior of polymers exposed to particular environmental conditions through the use of accelerated aging tests, international commissions (International Organization for Standardization–ISO, European Standards–EN or the American Society for Testing and Materials–ASTM) have proposed different standards describing such procedures [23,24,25,26]. Almost none of these standard procedures, however, indicates that a precise correlation factor between the results obtained from natural exposure and those from accelerated aging exists.

On the contrary, some of them expressly report that accelerated aging procedures cannot be exactly correlated with natural exposure: the results obtained by the standard procedures cannot be considered equivalent to those of any outdoor aging test, unless the degree of quantitative correlation has been calculated for the polymer under analysis. Furthermore, generally they do not impose strict conditions on how accelerated procedure must be performed, in particular on the duration of the tests in order to provide reliable results: this choice is often left to the operator’s experience, although guidelines are provided based on the type of application/exposure level. Some important aspects, finally, are neglected in the standard codes, such as the chemical nature and the composition of a polymer, both playing important roles in the stability of a polymeric material against any external agent.

## 3. Comparison between on Field Durability Tests with Accelerated Procedures

**Figure 3:**
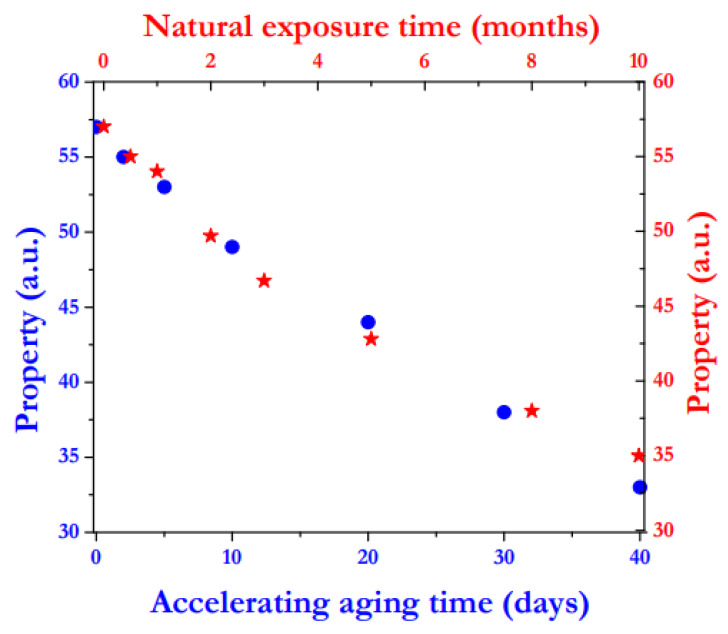
Correlation between the values of an arbitrary property measured on a polymer outdoor exposed and those recorded for the same polymer subjected to an accelerated aging procedure, with the aim to calculate an “Equivalent time”.

The rationale for the adoption of accelerated aging procedures clearly resides in the possibility to make reliable previsions of the on-field long-term durability of a polymer employing fast laboratory tests. This would imply the existence of good correlations between natural exposure tests and accelerated aging procedures. It could be stated, therefore, that the accelerated aging tests would be truly useful to make realistic durability previsions only if a precise correlation between the real behavior of the material exposed to the true external environment, whatever it is, for prolonged times and the experimental data from the accelerated aging tests would exist; in other words, if an “Equivalent time” can be calculated for the polymer under analysis, as in a hypothetic study illustrated in Figure 3. In other studies, an accelerated factor (*k*) is defined, as:

(1) $$k=\frac{t_{outdoor\;exposure}}{t_{accelerated\;aging}}$$

where: *t_outdoor exposure_* and *t_accelerated aging_* are the times necessary to achieve the same value of the property under study after natural exposure and accelerated aging process, respectively [27].

On the other hand, no unique correlation factors between results from accelerated procedures and natural exposure are available to date due to the fact that: UV and solar spectral radiations somehow differ; the dosage of the exposure parameters (temperatures and thermal variations, presence and level of moisture, UV-solar radiations) depends, in the case of outdoor exposure, on the geographic location and on the season, and they can vary from day to day, as already underlined; complex interactions can occur between the different parameters. Moreover, in some standards or widely adopted practices, too severe conditions, not always comparable with true on-field exposures, are employed leading to un-realistic results. Some examples are following presented for the most common commercial polymers.

The identification of the more suitable procedure to make reliable previsions of long term performance of outdoor exposed greenhouse covering films has been the subject of several papers [28,29]. In agricultural applications, the polymeric films, mainly based on low-density polyethylene (LDPE), are continuously and severely weathered for prolonged times, especially under the concomitant effect of UV radiations and oxygen; any degradation/modification of the films, on the other hand, can appreciably affect the quantity and quality of the crops produced inside the greenhouses. Therefore, the main purpose of such studies is the assessment of the effects of the introduction of additives (antioxidants, UV-stabilizers, hindered amine light stabilizer, etc.), capable to improve the durability of the PE films by retarding the onset of degradative phenomena occurring usually after two-three years of natural exposure [29]. To this regard, the possibility to test the durability of a LDPE film through an accelerated aging procedure allows to identify the best modified composition in times substantially shorter if compared to the typical on field observation periods not suitable for making industrial choices. The reviewed studies, carried out by two independent research groups, confirmed that significant different degradation mechanisms took place in polyethylene films upon exposure to natural weathering or to accelerated procedures. These latter provided for prolonged exposure to a lamp at 340 nm [29] or to an UV-B type lamp in a QUV environmental chamber and to UV mercury lamp [28]; in both cases, the test temperature during the accelerated aging procedures never exceeded 50 °C. Different effects were observed on the natural weathered or artificially aged LDPE films in terms of mechanical properties, rheological behavior, transparency of the films [28]. In this study, it was not possible to calculate a precise equivalent time from the results obtained on weathered or artificially aged specimens since the accelerated procedures provided a general overestimation of the degradation effects caused by outdoor exposure. In the other study [29], the equivalent time for accelerated aging with respect to natural exposure was calculated for the tensile mechanical properties on each of the different LDPE compositions analyzed: it was found that the calculated equivalent times varied from one formulation to another. The data collected from Fourier Transform Infrared (FTIR) spectroscopy, performed on the films aged under natural or accelerated conditions, revealed different characteristic chemical groups formed after the two different exposure regimes, the natural outdoor exposure (performed in Riyadh, Saudi Arabia) producing much more severe effects than accelerated exposure.

Polypropylene (PP), employed in many different outdoor applications, is greatly affected by weathering, and particularly by radiations that produce modification in mechanical properties, particularly embrittlement, and discoloration [30]. PP and its composites containing inorganic talc were naturally and artificially aged, highlighting the modifications on polypropylene due to the different aging procedures [21]. While the outdoor exposure of PP was carried out for one year in Japan, three accelerated aging procedures were employed with the aid of xenon (120-h cycle), metal halide (6-h cycle) and carbon arc lamps (60-h cycle), always performing the aging at 63 °C. The accelerated aging cycles included also the presence of water spray, in the case of the apparatus with xenon or carbon arc lamps, or of condensation water for the last testing machine. The Author of the study found appreciably different degradation levels in PP exposed to natural or accelerated aging procedures, this result attributed to the different features and magnitude of the environmental agents characterizing natural exposure or accelerating aging, respectively.

**Figure 4:**
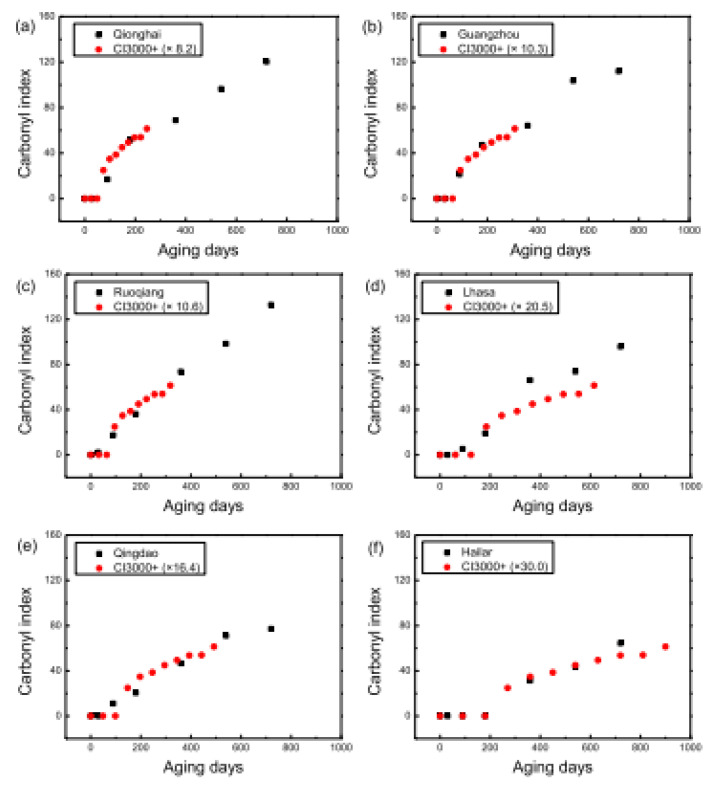
Comparison between results from outdoor exposure, performed in six different locations in China ((**a**): Qionghai; (**b**): Guangzhou; (**c**): Ruoqiang; (**d**): Lhasa; (**e**): Qingdao; (**f**): Hailar), and predictions from accelerated aging, based on carbonyl index. Reprinted with permission [31].

The calculation of the correlation factor between natural durability of outdoor exposed isotactic polypropylene (iPP) and the durability of the same polymer subjected to an accelerated aging procedure was the subject of the research published by Lv and co-workers [31]. The natural weathering experiments were carried out for two years in six locations in China, representative of different climatic conditions. The accelerated aging procedure, based on the code [32], was performed with the aid of a Xenon lamp, equipped with borosilicate filters, in order to reproduce as close as possible the solar radiations in the ultraviolet and visible regions. Apart from a continuous irradiation, wet (spraying deionized water on specimen’s surface) and dry (at 65% of relative humidity, *RH*) cycles were accomplished at a temperature of 55 °C. The results collected from spectroscopic (FTIR), calorimetric (Differential Scanning Calorimetry, DSC) and molecular weight (Gel Permeation Chromatography, GPC) analyses, observations from Scanning Electron Microscopy (SEM) and data from tensile tests suggested that the accelerated aging procedure employed was able to reproduce to a certain extent the main effects due to the outdoor exposure of iPP. The wide study demonstrated also that the correlation time between accelerated aging and outdoor exposure substantially depend on the natural weathering conditions, ranging from 8 for the most severe environmental conditions to 30 times for the less severe ones, as illustrated in Figure 4.

Polypropylene is also widely employed in addition with fillers of fibers to produce particulate composites or fiber reinforced polymers (FRP) for diverse (automotive, construction and building, furnishing, etc.) applications. The use of natural fillers-fibers, such as wood flour, hemp or jute, instead of inorganic fillers, glass/carbon fibers gives these bio-composites a sustainability profile. In this context, bio-composites based on PP reinforced by hemp fibers were naturally and artificially aged in order to correlate the outdoor and accelerated weathering effects on different (chemical, thermal, mechanical, surface, morphological) properties of the bio-composites and to calculate an equivalent time corresponding to the same degradation due to the different aging procedures [33]. The outdoor exposure lasted one year and it was accomplished in France, in a South West region. The accelerated aging was performed according to the code [26], with the aid of a fluorescent UVA-340 lamp, alternating UV exposure dry cycles with hot (at 50 °C) condensation cycles and short water spry steps, up to an exposure time equal to 1000 h. Both natural and accelerated regimes brought about a degradation of the PP composite as well as of the polypropylene matrix, even though the outdoor exposure was found to be more detrimental for mechanical behavior of hemp-reinforced PP while polypropylene in isolation was most affected by the accelerated aging. Taking into account the diverse properties measured after different time spans on the materials exposed to the two aging procedures, the Authors of the study calculated, employing a statistical analysis, an acceleration factor able to reproduce the effects of outdoor exposure with the selected artificial aging. However, the acceleration factor was found to be strongly dependent on the type of material under analysis: i.e., a one-year natural exposure of un-reinforced polypropylene was well reproduced by 250 h of accelerated aging; on the other hand, the acceleration factor was much greater, equal to 750 h, in the case of the hemp-PP composite. Similar results were found in another study [34] where is was observed that bio-composites, based on PP reinforced with wood flour, were more severely degraded under natural exposure, performed in South of France for one year, than if subjected to an accelerated aging. This latter was performed with a xenon-arc lamp (wavelength range 300–400 nm), alternating cycles of UV exposure at 60 °C and 65% of RH with cycles of spray water.

In order to use recycled polyethylene terephthalate (PET) to manufacture new plastic products, the degradation occurring in post-consumer plastic PET bottles was analyzed [11]. The Authors of this study, furthermore, tried to correlate the results of accelerated aging and natural exposure (performed in London, U.K.) to predict the service life of the outdoor exposed PET items. The selected accelerated procedure consisted of repeated cycles of exposure to UV radiation at 60 °C for 8 h, followed by a condensation step at 40 °C for 4 h in absence of irradiation, up to 13,000 h of exposure. The accelerated aging was performed in a QUV chamber according to a proper standard [26], employing UV-A lamps (340 nm, power of 0.68 W/m^2^) considered to correspond fairly well to the noon summer sunlight in Northern European Countries. Impact strength and superficial characteristics (i.e., color and gloss) were the properties analyzed on PET after accelerating aging or natural exposure. No correlation among the two aging regimes was found for impact strength results: naturally weathered material (exposed up to 13,000 h) displayed only plastic deformation without fracturing; conversely, UV aged PET samples sowed a brittle failure even if exposed for only 250 h, with a consequent dramatic reduction in impact strength (about 85% from 9000 h of exposure onwards). The data from color measurements recorded on the aged specimens during the two (natural and accelerated) procedures, on the other hand, allowed to conclude that it is possible to reproduce the aging due to one year of natural exposure with about 25 days of UV accelerated aging, this correlation being applicable only for this characteristic.

The well-known engineering thermoplastic polycarbonate (PC) is widely employed in various outdoor demanding applications, to produce automotive and aeronautic parts, for building and construction components. It is reported that PC degradation originates from photo-Fries rearrangement reactions and/or processes of photo-oxidation [35]. Upon the creation of chromophores and modification in macromolecular structure (i.e., breaking of macromolecular chains and/or formation of cross-links), the (optical, mechanical) properties of the polymer are altered. Un-protected bisphenol A polycarbonate (BPA-PC) was weathered employing both outdoor natural exposure (in France, at the Atlas Sanary sur Mer Test Service Site) and accelerated procedures, analyzing, in particular its aging rate [36]. Different correlating factors between the two procedures were calculated (from 630 to 980 h of accelerated aging corresponding to 1 year of natural exposure) depending on the technique employed to quantify the degradation rate, i.e., UV absorption or IR-absorbance, respectively. In a more recent study [13], a bisphenol A polycarbonate, intended for optical applications, was exposed to accelerated tests involving high intensity blue light and elevated temperatures (ranging from 90 to 120 °C), to make service-life predictions of the reliability of PC exposed to milder use conditions. The measured property in this case was the discoloration degree of the polycarbonate, calculated as yellowness index with the aid of a spectrophotometer. The photo-degradation kinetics of PC resulted very different for accelerated aging procedure and outdoor weathering, suggesting the occurrence of different degradation mechanisms.

**Figure 5:**
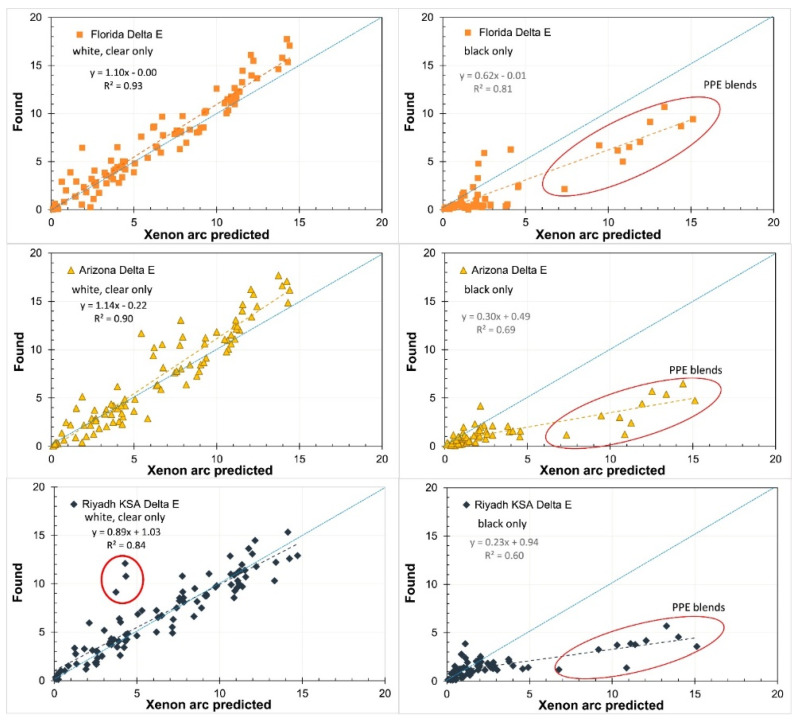
Found vs. predicted color shifts for white and clear materials (**left**) and black materials (**right**). The blue dotted lines show slope = 1 while the colored dashed lines show the linear regression with the equation in the legends. The three circled points on the lower left plot occurred near an abrupt color shift. The circled points on the right side graphs show the PPE blends. Reprinted with permission [18].

In a recent wide research work, several (i.e., 31) aromatic engineering thermoplastic polymers were outdoor exposed during two years in different (i.e., 4) sites (in U.S.A. and Saudi Arabia) [18]. The same materials were subjected to an accelerated aging procedure designed modifying a standard procedure [32]. For this latter, xenon arc lamp was employed with the aim to identify the polymer’s properties whose behavior upon outdoor exposure can be almost precisely predicted by accelerated weathering procedures employing a standard protocol. Depending on the analyzed property/characteristic (tensile mechanical, Charpy impact resistance, color and gloss, surface erosion, transparency), and on the place of natural exposure, the Authors of the study identified a good or, on the contrary, a limited reproducibility of the effects of natural exposure with those obtained with the selected accelerated aging, as illustrated in Figure 5. The reproducibility was scarce especially in case of particular environmental conditions, such as sand and wind erosion in Saudi Arabia, affecting some of the properties of the materials and whose effects cannot be simulated with a xenon lamp.

The possibility to simulate with accelerated aging procedures the effects due to natural weathering on styrene-ethylene-butylene-styrene (SEBS) copolymers was the subject of the work presented by White and co-workers [37]. The accelerated aging was accomplished with a UV lamp with wavelength in the range 295–450 nm, exposing at the same time the specimens to different temperature and humidity levels, i.e., 30° and 55 °C, <1% and 80% *RH*, respectively. FTIR data, in terms of the rate of photo-oxidative process measured on the copolymer exposed to accelerated aging, were compared with those collected on SEBS samples after outdoor exposure at Gaithersburg, Maryland State (U.S.A.). The photo-oxidation was found to be strongly depend on the accelerated aging temperature and less affected by the humidity level. Accordingly, naturally weathered SEBS copolymers displayed a much slower photo-oxidation than the same copolymers aged at a temperature (80 °C) much greater than that maximum achievable in true external conditions, i.e., 46 °C. The acceleration factor in comparison to the outdoor exposure was found to depend on the parameters employed in the selected accelerated aging procedure, ranging from 2.5 to 10 times.

In a similar study [38], the photo-oxidative degradation occurring on acrylonitrile-butadiene-styrene (ABS) polymer as a consequence of outdoor exposure or accelerated aging procedures was analyzed with the aim of calculating the correlation factor between the two aging regimes. The effects of photo-oxidation were assessed through FTIR analysis and mechanical properties measurements. The results, found in a good agreement, allowed to conclude that the exposure for 1260 h to a filtered xenon lamp (wavelength range 300–400 nm), at 48 °C and 50% *RH*, was able to satisfactorily mimic the effects of one year of outdoor exposure carried out in Lisbon.

Surface treatments based on polymers, known as protective coatings, are commonly applied to different substrates (metals, stone, wood, etc.) to limit their degradation mainly due to corrosion, water ingress, UV damages, biological attack, preserving the properties and the appearance of the substrate. The selection of the most appropriate coating, in terms of composition, features and performance, as well as the most suitable application techniques depends on many factors, such as the nature and characteristics of the substrate, the exposure site and climate, specific requirements. Once applied to the substrate to protect, it is essential to analyze the durability of the coating, either by natural exposure and accelerated aging, to assess after how long its performance decays, and therefore it must be replaced [39,40,41].

The development of an accelerated aging protocol able to reproduce the degradation observed upon natural exposure, performed for two years in South Florida, in polymeric coatings for automotive and aerospace applications, was the aim of the study carried out by [14]. This area is characterized by environmental conditions particularly harsh, due to the combined action of intense solar radiations and prolonged and high humidity levels, leading to severe degradation processes (UV induced photo-oxidation, moisture induced hydrolysis, surface erosion). The designed aging procedure, therefore, involved the exposure to several alternating wet/dry, dark/light cycles, with different durations. It was found to be able to appreciably reproduce the effects due to natural weather characterizing South Florida on the main properties of polymeric coatings for automotive and aerospace applications. However, the acceleration factor of the new aging protocol was dependent on the kind, thus the application, of analyzed coating, with a greater acceleration factor for automotive coatings and a smaller acceleration for aerospace ones.

When analyzing the durability of polyurethane/polysiloxane hybrid coatings, accelerated aging procedures were found to produce much more severe effects than three outdoor harsh exposures (i.e., industrial, volcanic and marine environments) [42]. The accelerated aging was performed in a weather-meter machine for 3 months, exposing the specimens to light (with the aid of a Xenon Arc lamp), moisture (spraying water in a “dark” cycle) and increasing temperature to mimic the morning conditions. The Authors of the study, investigating the chemical structure (with FTIR and Nuclear Magnetic Resonance, NMR, analyses), the thermal properties (employing Dynamic Mechanical Thermal Analysis, DMTA, DSC and Thermo-Gravimetric Analysis, TGA) and the coating morphology (through a SEM), did not report any correlating factors in the results between the different kinds of natural and artificial aging exposures, due to the substantially different effects produced.

The durability of a nanostructured organic-inorganic (O-I) hybrid coating for stone protection due to the exposure to natural or accelerated aging processes was investigated by [43]. The accelerated aging was performed according to the code [44] exposing the coating, once applied to the stone surface, to repeated cycles consisting of an irradiation step (exposure to UV-A (340 nm) lamp at 60 °C) alternated with a water condensation step at 50 °C; the total duration of this procedure was 4 months. The effects to natural exposure were evaluated in Lecce (South Italy) with an observation period of two years. The properties evaluated after the different aging procedures were hydrophobicity capability and the color variation, both being primary parameters for evaluating the effectiveness of a surface treatment for stone protection. The comparison of the results from contact angle measurements and of color change data, collected after outdoor exposure and artificial aging procedure, allowed to calculate a correlation time-factor found to be similar for these two characteristics, i.e., the exposure to accelerated aging for 20 days corresponded to 730 days or outdoor exposure.

Thermosetting polymers (such as epoxy, acrylic, vinyl ester resins) are frequently used as adhesives, coatings, matrices for composites: since in all the mentioned applications the materials are outdoor exposed and subject to more or less severe weathering, the possibility to obtain reliable predictions of their long-term performance with accelerated aging procedures represents an asset [45,46,47,48]. Durability studies appeared in literature on the effects caused on thermosetting matrices for composites by artificial and natural weathering have shown that even in the case of this class of materials it is not possible to obtain an adequate correlation of the test results from natural exposure and accelerated aging procedures.

Belec and co-workers compared the effects of natural weathering and artificial aging procedures on an epoxy-based composite containing unidirectional glass-fibers [49]. The accelerated aging procedure, carried out up to 8 weeks, was based on irradiation by a fluorescent lamp (at 340 nm), in presence of a test temperature equal to 45 °C, without any condensation step. The specimens were separately exposed to hygrothermal aging conditions, at a RH of 85% and a temperature of 70 °C. Tropical environmental conditions were selected for the natural aging, i.e., exposing the specimens up to around one and half years (more precisely 76 weeks) in Danang (Vietnam). The Authors of the study identified as primary effects of natural and accelerated aging the plasticization of the amine-cured epoxy matrix, due to the moisture characteristic of both tropical environment and hygrothermal aging, and scissions of polymeric chains due to natural and artificial UV exposure. However, no correlation time-factor was proposed in their study.

Fiber reinforced composite elements, intended as reinforcements for civil engineering infrastructures, based on unidirectional carbon fibers and a vinyl-ester matrix, were subjected to different outdoor exposure (four locations in Portugal, characterized by diverse environmental and pollution conditions, employing a two years exposure time) and accelerated aging procedures (i.e., immersed in water at 20 °C or collecting data from previous studies reported in literature), comparing the effects of the different weathering regimes [50]. The Authors of the study reported that the accelerated aging protocols generally caused a greater degradation in mechanical (measured in tensile mode) properties, especially the tensile strength, with respect to the exposure to natural aging, irrespective to the exposure site. They concluded, furthermore, that their efforts to identify a correlation between the effects on CFRP due to natural exposure and accelerated aging procedure failed and suggested longer natural weathering times for future investigations.

**Figure 6:**
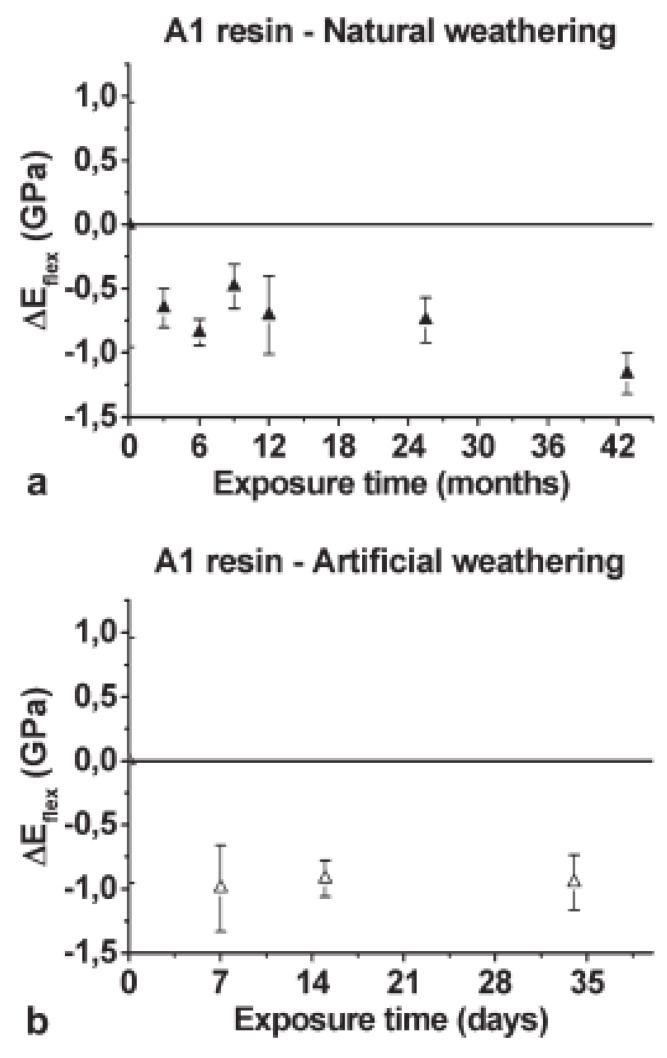
Variations in the flexural modulus of elasticity (*ΔE_flex_*) measured on A1 resin relative to the initial value calculated for unexposed specimens measured during (**a**) natural weathering and (**b**) artificial aging. Reprinted with permission [52].

In the same study, the durability of two cold-cured epoxy adhesives was also investigated, trying to compare the effects of natural exposure to those due to accelerated aging protocols reported in literature. Cold-cured structural epoxy resins are, in fact, the adhesives typically employed in civil engineering applications to apply a FRP element on the surface to strengthen or as matrices for in-situ formed FRPs; they display peculiar characteristics, such as very long curing times to achieve a not complete conversion degree of epoxy groups and a glass transition temperature never exceeding 65–70 °C [51]. Even for this class of polymeric materials, the mechanical data collected after natural or artificial aging regimes were found to be not comparable, the accelerated ageing tests leading to greater reductions in mechanical properties if compared to the effects due to natural exposure [50]. Similar results have been previously reported by [52] that tried to correlate the effects of natural exposure (carried out in two different locations in South Italy) and artificial aging on thermal (i.e., Tg) and mechanical (in flexural mode) characteristics of cold-cured epoxy adhesives. Although qualitatively similar changes in thermal and mechanical properties were recorded as consequence of natural and artificial agings, the selected accelerated procedure resulted excessively severe if compared to the aging caused by outdoor exposure, as illustrated in Figure 6.

From this overview, the following conclusions can be drafted: (i) there is no uniformity of tests conducted even on the same class of polymers, different researchers perform accelerating tests using different standards, setting different exposure parameters and with different devices, checking the variation of different properties (mechanical, surface, thermal, etc.); (ii) there is no uniformity between the observed results, some scholars find correlation times, others do not; (iii) the correlation times, if calculated, depend on the polymer being analyzed, on the apparatus and testing conditions, on the properties measured on it and on the place where its outdoor exposure is carried out. From what has just been observed, one of the key parameters of such procedures seems to be the setting of proper accelerated aging conditions: these latter must take into account from one side the outdoor exposure that must be reproduced and from the other the kind of polymer under investigation. Furthermore, the effects due to natural exposure can be time-of-year dependent since the natural solar radiation depends qualitatively and quantitatively on the exposure season of the polymeric material. Thus, the correlation factors should also take into account the time of year in which the natural exposure was conducted. Referring to the kind of polymer, the aging conditions must be selected in order not to induce unlikely chemical reactions/physical processes.

To answer the question of the title of this work, accelerated aging tests could be a useful tool to predict the long term behavior of polymers exposed to different environments but they still require optimizations in terms of: development of suitable devices; setting of appropriate aging parameters and times, that can both depend on the kind of polymer; clear indications of the characteristics/properties to be measured, how to analyze the results and how to calculate correlation factors with natural exposure data. All these needs should be met by updated codes.

## 4. Relationships and Models

From the previous examples, it is clear that the key role of such investigations, as well as their practical usefulness in predict the lifetime of a polymer, relies in the identification of a mathematical relationship between the behavior of the polymer subjected to either outdoor exposure and accelerated aging procedure [53]. Since, as already underlined, polymers during their service life are simultaneously exposed to different and highly variable environmental factors (radiations of various wavelengths and light intensity, heat, oxygen, water, relative humidity being the most common ones), the identification of the proper correlation between natural and accelerated aging procedures for each polymeric material is a challenging goal.

To this regard, the well-known Arrhenius model has been widely employed to predict the long-term behavior of polymers, hence, their reliability in service [13,38,54,55,56]. This model is employed when the accelerating parameter is selected as the temperature of the aging procedure: in this way, it is possible to test the polymer at temperatures much larger than ordinary ones in order to promote degradation in a shorter period of time than that required for the material to achieve the same degradation at room temperature [57].

For testing over some range of accelerating variables, one can fit a model to the data to describe the effect that the variables have on the failure-causing processes. For some situations, a physically reasonable statistical model may allow such extrapolation [58].

It is important to highlight that the assumption of constant temperature, thermally activated lifetime, based upon the Arrhenius assumptions, does not always provide the necessary understanding to interpret accelerated tests [59]. Several issues arise on the applicability of this procedure to cover a wide range of temperatures: as already pointed out, in fact, variations in the physical state of the polymeric material could occur as a consequence of the approach to the Tg or Tm range of the polymer. This is the case, for example, of the aforementioned cold-cured epoxy resins which fail to achieve 100% conversion of the epoxy groups during the curing process: the aging procedure at temperatures above the moderate *Tg* of the resin would, therefore, cause a post-curing process in the resin. This heat treatment could change the way the material degrades under true service conditions, making the predictions obtained with the Arrhenius model unreliable and unrealistic. Therefore, the good fit between analytical model and the real results will depend on the good selection of parameters, the right data correlation, the right understanding of the science of failure as well as the degradation mechanism affected by the accelerated test.

## 5. Conclusions

Although many investigations have been carried out over the years to reproduce the effects on polymers due to natural exposure using accelerating procedures, thus reducing analysis times, discrepancies were generally observed between on-field tests and accelerated ones due to the ineffectiveness of the latter to reproduce the effects of complex weather conditions, as highlighted in the present work. To reproduce the effects of natural exposure in short observation times, the acceleration factors are frequently excessively amplified, making the results unrealistic. Furthermore, if from one side polymers, i.e., a wide class of materials, can be not equally affected by the same environment, from the other the environmental conditions changes from one region to another: as a consequence, it may be necessary to define different standards for each type of polymer and, possibly, for each climatic zone. Nevertheless, even though accelerated aging procedures cannot be straight employed to make reliable previsions on the long-term behavior of a polymer, exposed outdoor or to a specific environment, the results of such tests can be of undoubted utility for a qualitative assessment of the weathering effects, and used as an upper reference usage limit.

The analysis of the recent literature on this topic allows to provide some points on which the investigations should be mainly focused:

(1) The identification of proper analysis times as well as thresholds for the acceleration factors (in terms of temperature, UV radiations, relative humidity, etc.) that must not be exceeded in order not to trigger phenomena that would not occur in real conditions; such parameters could even depend on the specific polymer and on the outdoor exposure that must be reproduced;
(2) The updating of the standard codes reporting clear indications of the characteristics/properties to be measured, how to analyze the results and how to calculate the correlation factor with natural exposure data.

It is, finally, advisable to collect and rationalize experimental results from different studies, in order to have a large amount of data to process with empirical correlation, in order to be able to predict long-term behavior of any commercial polymer.

## Data Availability

The study does not report any new data.
